# Authentication of Smartphone Users Based on Activity Recognition and Mobile Sensing

**DOI:** 10.3390/s17092043

**Published:** 2017-09-06

**Authors:** Muhammad Ehatisham-ul-Haq, Muhammad Awais Azam, Jonathan Loo, Kai Shuang, Syed Islam, Usman Naeem, Yasar Amin

**Affiliations:** 1 Faculty of Telecom and Information Engineering, University of Engineering and Technology, Taxila, Punjab 47050, Pakistan; awais.azam@uettaxila.edu.pk (M.A.A.); yasar.amin@uettaxila.edu.pk (Y.A.); 2 School of Computing and Engineering, University of West London, London W5 5RF, UK; jonathan.loo@uwl.ac.uk; 3 State Key Laboratory of Networking and Switching Technology, Beijing University of Posts and Telecommunications, Beijing 100876, China; 4 School of Architecture, Computing and Engineering, University of East London, London E16 2RD, UK; syed.islam@uel.ac.uk (S.I.); u.naeem@uel.ac.uk (U.N.)

**Keywords:** activity recognition, behavioral biometrics, continuous sensing, micro-environment sensing, mobile sensing, smartphone authentication, ubiquitous computing

## Abstract

Smartphones are context-aware devices that provide a compelling platform for ubiquitous computing and assist users in accomplishing many of their routine tasks anytime and anywhere, such as sending and receiving emails. The nature of tasks conducted with these devices has evolved with the exponential increase in the sensing and computing capabilities of a smartphone. Due to the ease of use and convenience, many users tend to store their private data, such as personal identifiers and bank account details, on their smartphone. However, this sensitive data can be vulnerable if the device gets stolen or lost. A traditional approach for protecting this type of data on mobile devices is to authenticate users with mechanisms such as PINs, passwords, and fingerprint recognition. However, these techniques are vulnerable to user compliance and a plethora of attacks, such as smudge attacks. The work in this paper addresses these challenges by proposing a novel authentication framework, which is based on recognizing the behavioral traits of smartphone users using the embedded sensors of smartphone, such as Accelerometer, Gyroscope and Magnetometer. The proposed framework also provides a platform for carrying out multi-class smart user authentication, which provides different levels of access to a wide range of smartphone users. This work has been validated with a series of experiments, which demonstrate the effectiveness of the proposed framework.

## 1. Introduction

Smartphones are ubiquitous, becoming more and more sophisticated with the advancement in their computing, sensing, and networking powers. Currently, 68% of the world’s population own a mobile phone, and by 2019 this figure is expected to be in the region of 72% [1]. Market research on the sale of smartphone has shown that the number of smartphones sold has surpassed the number of laptops sold worldwide [2]. The pervasive nature of smartphones, together with integrated sensing capabilities, has changed the landscape of people’s everyday life. Smartphones have become the guardians for most of our personal information, such as medical information (e.g., heart rate), bank account details, and personal credentials for different services and applications. With the increasing use of smartphones, users have begun to worry about the confidentiality of their data and information. As smartphones are intended for quick and recurrent access, it can lead to compromised privacy of smartphone data and information [3]. It has now become critical to maintain the privacy of sensitive data and information available through these devices using non-intrusive yet viable authentication mechanisms.

Unfortunately, most widely used authentication methods for smartphones including passwords, PINs, pattern locks, and fingerprint scans offer limited safekeeping [3], as they are vulnerable to many attacks including guessing [4], spoofing [5] in case of fingerprint scans, and the side channel attacks such as video capture attacks [6], or smudge attacks [7]. Secure passwords are often not considered to be appropriate for use on smartphones due to the length of time required for their input. Many smartphones provide PINs as alternatives to passwords. PINs have the benefit of being able to be entered quickly, but they provide far less safekeeping than passwords, as they may be guessed more quickly [3]. Pattern locks provide protection by allowing users to choose a sequence of points during enrollment and then repeating it during authentication. However, these pattern locks are exposed to side channel attacks, and the user’s fingertips often leave a distinguishing trace on the screen, which can indicate the pattern that was used to access the device [3]. Moreover, these authentication methods require a user to deal with the smartphone actively and spend a few precious seconds for inputting some valid pieces of information, or drawing sophisticated patterns on touchscreen, which has become a frustration for the millions of smartphone users worldwide. As a result, many people like to use fewer privacy barriers each time they decide to access their device [8], which is reducing the effectiveness of such authentication schemes, ultimately making them vulnerable to data theft. In addition, these commonly used methods for authentication fail to detect and recognize an adversary once he/she has passed the point of entry [9], which makes these approaches futile for continuous and non-intrusive passive authentication.

**Figure 1:**
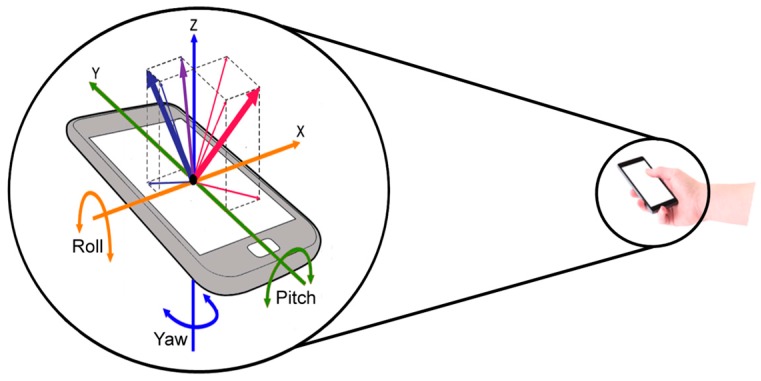
Smartphone inertial sensors are sensitive to the orientation of the smartphone. The accelerometer measures acceleration, the gyroscope measures rotation, and the magnetometer measures the magnetic field strength along the *x*, *y*, and *z* axes.

Continuous and passive authentication aims to address these challenges by offering a way to use behavioral biometrics for authenticating a smartphone user continuously [9]. Behavioral biometrics based authentication scheme targets to learn the characteristics of the user behavior that does not change over a period of time, such as gait patterns [10], hand movements and waving patterns [11], voice [12], signature [13], and touchscreen interactions [14,15]. These characteristics are then used to implicitly authenticate a smartphone user to prevent unauthorized access to the device. This type of authentication works passively in the background and monitors the interactions between a user and the device to make a decision about the authenticity of the user who is trying to use the device [9]. The user authentication decision is taken on the basis of distinctive features identified from the user’s behavior. Recent research has been exploiting smartphone inertial sensors for developing fast and secure authentication schemes based on behavioral biometrics [16,17,18]. A vector representation of the axes of smartphone inertial sensors is shown in Figure 1.

The research on behavioral biometrics is challenging because of the difficulty of collecting data from a practical and legal point of view [18]. Existing research has found issues with data collection procedures due to inadequate amount and diversity of data, poor representation and description of real world events, and crucial self-consciousness of the members participating for performing different activities. In addition, other challenges associated with the development of a continuous authentication system for smartphones are as follows:

- Orientation sensitivity of smartphone inertial sensors, i.e., the readings of these sensors change by changing the orientation of the smartphone, as shown in Figure 1.
- Effectively learning activity and motion patterns from noisy sensors data.
- Incorporating real-time sensors data into a biometric authentication setup on a smartphone, which is limited in terms of memory and processing power.
- Lack of “negative” samples for efficient testing of an authentication model.

Keeping all these issues and challenges in view, the problem of continuous and passive authentication of a smartphone user is addressed in this study. A novel Intelligent Authentication (IntelliAuth) scheme is proposed for smartphone user authentication, which is based on physical activity recognition and micro-environment sensing. The activity recognition component is based on recognizing behavioral patterns from a series of activities performed by the user, while micro-environment sensing is based on recognizing elements within proximity of the surrounding area of the mobile phone [19] For the purpose of user authentication, six Activities of Daily Living (ADLs) are considered in this study: walking, sitting, standing, running, walking upstairs, and walking downstairs. Three smartphone sensors, i.e., accelerometer, gyroscope, and magnetometer, are used for capturing data of different smartphone users while performing these activities. As the position of a smartphone on the user’s body may vary while performing any activity in real time, therefore, five different positions are chosen for placing the smartphone on the user’s body while performing one of the six defined activities. These body positions include right wrist, right upper arm, left thigh, right thigh, and waist position towards right leg. A smartphone is supposed to be placed by the user in one of these body positions while performing an activity in real time.

For validation, an existing dataset for physical activity recognition [20,21] is utilized. The data are pre-processed, and several features are extracted from time and frequency domains. The extracted features are then classified into six different activities performed by three different classes of smartphone users. Three different user classes selected in this study are authenticated, supplementary, and impostor. Each user class symbolizes a smartphone user having a different level of access to smartphone data. Four different classification algorithms, i.e., Support Vector Machine (SVM), Bayesian Network/Bayes Net (BN), Decision tress (DT), and K-Nearest Neighbors (K-NN), are employed for activity classification. A probabilistic scoring model, based on activity recognition, is used to classify a smartphone user for the purpose of authentication.

The primary contributions of this research work are:

1. A novel and multi-class smartphone user authentication scheme, based on activity recognition, is presented for different types of users that may access a smartphone.
2. Micro-environment sensing is combined with physical activity recognition to eliminate false positives arising due to the position sensitivity of smartphone inertial sensors, resulting in better user authentication.
3. A novel probabilistic scoring model, based on activity recognition, is presented for smartphone user classification.

The rest of the paper is structured as follows: Section 2 presents a brief description of the related work. Section 3 provides a detailed description of the IntelliAuth framework for user authentication. Section 4 explains the methodology used in this research work for activity recognition and smartphone user authentication. In Section 5, a detailed analysis of the results is presented and discussed. Section 6 concludes the research findings, and provides recommendations for future work.

## 2. Related Work

As computing and sensing capabilities have advanced in smartphones, researchers have started to utilize more types of sensory data from these devices for a wide range of purposes. Mobile sensing data have been exploited for crowdsourcing [22], context awareness [23,24], and activity recognition [25]. Existing work shows that utilization of multiple on-body sensors placed at different positions (i.e., waist, knees, arms, and ankles) can determine the physical activities performed by a user [26,27,28]. In [29] and [30], data pre-processing and feature extraction algorithms were applied for activity recognition using an accelerometer. In [31], the authors detected complex human activities such as smoking, eating, drinking, etc. by utilizing smartphone sensors along with wrist-mounted motion sensors. Activity recognition has been utilized for different purposes, such as human behavior modeling [32,33] and health monitoring [34]. The authors of [35] applied activity recognition techniques for detecting bad habits in a person by combining smartphone sensors with wrist-worn smartwatch sensors.

In a few recent years, the research on smartphone user authentication has seen determined work, and many solutions have been proposed for the authentication of smartphone users. A comprehensive review of the state of the art for smartphone user authentication is provided in [9], which lays emphasis on seven different behavioral biometric approaches for user authentication. These approaches include gait, touchscreen interaction, hand waving, keystroke pattern, voice, signature, and behavioral profiling. Zheng et al. [15] utilized accelerometer, gyroscope, and touchscreen sensor for non-intrusive authentication of a smartphone user by analyzing how a user touches the phone. Different features such as acceleration, pressure, size of touch area, and passage of time were collected using experimental data on both four-digit and eight-digit PINs by employing tap behaviors to verify passcodes of different participants. The authors used one-class classifier [36] based on the notion of nearest neighbor distance for user recognition. Trojahn and Ortmeier [37] proposed a scheme that combined keystroke and handwriting analysis on smartphones for the purpose of user authentication. During data recording, the authors asked different subjects to type a sentence or a password for a specific number of times. For evaluating their approach, the authors chose different machine learning algorithms including Decision Tree [38], Bayes Net [39], and MLP [40]. The authors in [18] proposed a scheme for on-device authentication of smartphone users by learning their motion patterns based on two essential components: time-based feature extraction using deep neural networks, and classification via a probabilistic reproductive model. Table 1 provides further existing work related to behavioral authentication of smartphone users by providing a comparison among different studies on the basis of the approach used for behavioral biometrics, classification algorithms, and the set of features employed for user authentication. The problems and limitations of different behavioral biometric approaches that have been used in the existing studies for smartphone user authentication are described in Table 2.

A few researchers [11,14,57] have concentrated on learning some specific activities for smartphone authentication—for example, picking up a smartphone from the table, unlocking the home screen using a slide pattern, dialing a specific number from the keypad, making a hand waving gesture, or making a call. However, these activities are specifically related to a smartphone and not proficient to use for continuous authentication. The reason is that these activities authenticate a user only when he/she performs one of these activities in a specific pattern. Once a user has been authenticated, there will be no other way to monitor the user unless s/he performs one of these specific activities again. It is possible that a smartphone may get stolen by an impostor while it is unlocked. In that case, the authentication model will not be able to know that the smartphone is possessed by an impostor until and unless a mismatching pattern is detected related to a specific activity. Also, there can be instances where a smartphone may get snatched while a person is talking on the phone. In such cases, this type of authentication model will fail to correctly identify a smartphone user continuously.

In this study, the problems and limitations of the existing approaches for smartphone user authentication have been analyzed, and an effective solution has been provided for passive and continuous authentication of smartphone users. The proposed scheme combines micro-environment sensing with physical activity recognition for authenticating smartphone users, incorporating context awareness.

## 3. IntelliAuth Framework

The basic purpose of a smartphone authentication scheme is to differentiate between an authorized smartphone owner and unauthorized individuals. This type of authentication relates to a binary-class user classification problem where a person who is the legitimate user of a smartphone is classified as authenticated, whereas all other persons are classified as non-authenticated. This limits the access of a smartphone to only a single authenticated user. However, in practice, it is seen that a smartphone is not only limited for use of a single person only. A smartphone owner may share his/her smartphone with a spouse, close friends, relatives, or colleagues for a variety of tasks, such as making a phone call, sending a text message, playing a game, watching a video clip, or even doing something auxiliary. However, the authorized user does not want any private information in the smartphone to be compromised, leaked, or stolen. A smartphone owner may want to allow a certain group of people to access only a few portions of his private data on the smartphone by retaining a different level of access to his/her smartphone data for different persons. Given this, any smartphone authentication framework, based on behavioral biometrics, will give rise to a lot of issues as the authentication framework will not be able to authenticate any person other than the original owner (authenticated user), and may not permit him/her to use that device at all.

In order to address the major challenges associated with the authentication of multiple smartphone users, the IntelliAuth framework classifies the smartphone users into three different classes: authenticated, supplementary, and impostor. This user classification is performed on the basis of activity recognition using a probabilistic scoring model. Being classified as authenticated user means that the user is the owner of the device and permitted to access all the data and information on the device. However, being classified as impostor means that the user is a fraud and should not be allowed to use that device at all. If the user authentication model finds a user as supplementary, it means that the user will gain only a restricted access to the smartphone as set by the owner of the device, i.e., the authenticated user. In short, the proposed framework assigns three different levels of access privileges, i.e., full-level access, restricted access, and zero-level access, to authenticated, supplementary, and impostor users of a smartphone, respectively.

The proposed framework utilizes a combination of three smartphone motion sensors, i.e., an accelerometer, a gyroscope, and a magnetometer, as a source of input data for activity recognition and user authentication. The use of a combination of the data from all three sensors is expected to improve the performance and accuracy of the user authentication process. Previously, in [20,21], it has been shown that the recognition accuracies of different activities can be significantly improved when multiple motion sensor data are combined, which is even more effective in the case of smartphones that are carried in different body positions. However, when there is less certainty surrounding smartphone positioning, a magnetometer used in a combination with an accelerometer and a gyroscope provides better accuracy for activity recognition. The study concluded that a magnetometer can be used in a combination with an accelerometer and a gyroscope to provide a supporting role for activity recognition. As the proposed framework for smartphone user authentication identifies a smartphone user by recognizing his/her activity pattern, a combination of these three sensors has been utilized in this study for the purpose of user authentication.

### 3.1. Recognition of ADLs for Smartphone User Authentication

Activity recognition is the building block in this research work, which is employed for user authentication. The proposed scheme primarily focuses on authenticating smartphone users by learning and recognizing their behavioral traits while using smartphone. For this purpose, six Activities of Daily Living (ADLs) are selected in this study. These activities include: walking, sitting, standing, running, walking upstairs, and walking downstairs. The motion patterns of these activities are learned for different classes of users. Generally, people perform these activities for multiple times on a daily basis, whether intentionally or not. Hence, a smartphone user whether authenticated, supplementary, or impostor, is likely to perform these activities in his/her routine life frequently. The proposed scheme authenticates a smartphone user on the basis of these activity patterns by continuously collecting and processing small chunks of sensors data in real time. The system recognizes the activity performed by the user from the collected chunk of data, and classifies the user as authenticated, supplementary, or impostor. If it finds the user to be authenticated, it permits the user to unlock the phone using a default option and access all the data and information on the smartphone. In the case of an impostor user, the system will not permit the user to unlock the phone at all. The system keeps on validating the user repetitively after a small interval of time, for example, five seconds, and takes no further action until a different class of user is identified. If an impostor user gets the smartphone while it is unlocked, the framework will identify the user within the five-second interval based on the selected activities, and the phone will be locked again. Hence, the ADLs mentioned above assist in providing better continuous and non-intrusive user authentication as these activities are based on tasks that are not only specific to the purpose of authentication but are performed by all smartphone users in general.

The proposed scheme validates and identifies a smartphone user based on the activity patterns for which the user authentication model is trained. For this purpose, the authentication model is trained to learn and recognize the motion patterns of the selected activities when performed by the users in a normal pattern as they tend to do usually. In real time, if an activity is performed by a user in a hefty random sequence or an abnormal pattern, whether intentionally or unintentionally, the authentication model is unlikely to be able to identify that smartphone user correctly. The key reason for failing to recognize the smartphone user is that the model is not trained to account for abnormal activity patterns of a user. Moreover, a random activity pattern of an authenticated user might be similar to the activity pattern of an impostor user. In that case, if the system is trained to adapt itself to the random activity patterns of an authenticated user, then the system may falsely accept an impostor as an authenticated user, thus erring towards the safety. However, besides offline training of the authentication model, the proposed framework allows the collection of sufficient amount of new training data for a user in real time. Thus, the model can be trained corresponding to the different motion patterns of the same activity performed by the same user. Training data can be collected for a new user as well, and a class label can be assigned to that user. The authentication model can then quickly learn the activity patterns for the new user from the collected data and adapt itself to the new user. In this way, the proposed framework also provides adaptive behavioral authentication.

### 3.2. Micro-Environment Sensing for Activity Recognition

A smartphone is not typically placed or kept at a single position only. A smartphone user may keep his/her phone at different body positions with different orientations while performing the same or different activity. The data collected from the smartphone inertial sensors is sensitive to the placement and orientation of the smartphone on the human body. The axes of the smartphone inertial sensors change their directions according to the orientation of the smartphone as shown in Figure 1. Hence, the readings of these inertial sensors also vary. In our daily life, we come across several people who keep the smartphone in their jeans while performing many activities, whether in the left jeans pocket or the right jeans pocket. A few people hang the smartphone by a clip attached at their belt at waist height, while others may keep the smartphone in a chest pocket or side pocket. Some people keep the smartphone in their hands most of the time while doing anything. A few people keep the smartphone at the upper arm position while doing activities like walking and running. Some females tend to keep their smartphone inside a purse or a small pouch hanging from their arm, normally at waist height. If a user changes the position or orientation of the smartphone on his/her body while performing an activity in real time, the readings of the smartphone inertial sensors will be different. Hence, the activity pattern will change. Thus, the authentication scheme will not be able to correctly identify the smartphone user on the basis of his/her activity pattern. This is one of the main challenges in creating an efficient scheme for smartphone user authentication based on activity recognition. The proposed scheme for smartphone user authentication addresses the issue of position sensitivity of the smartphone by incorporating micro-environment sensing [19], i.e., being aware of the close surroundings of the smartphone, with activity recognition for improved user authentication. For this purpose, five different body positions are selected in this study for placing a smartphone on the human body while performing an activity. These body positions are considered as the close surroundings of the smartphone, and include the left thigh, right thigh, waist, upper arm, and wrist position. The motion patterns of all selected activities are learned, corresponding to all user classes for these five positions of the smartphone on the human body. The user authentication model is trained to sense the position of the smartphone on the human body along with the activity being performed and the user who is performing that activity.

The positions of left and right thigh conform to the left and right jeans pockets on the front side, respectively, where a smartphone can be placed. The waist position relates to a belt clip above the right leg that can be used to hang a smartphone, or it may relate to the side pocket on the right side of a uniform. The wrist position is used to point out the presence of a smartphone in the hands, specifically in the right hand for this study. The upper arm position corresponds to an external phone holder attached to the right bicep, or may relate to a right side chest pocket. In [20,21], the authors also focused on these five body positions for placing a smartphone on the human body to recognize different activities.

## 4. Methodology of Research

**Figure 2:**
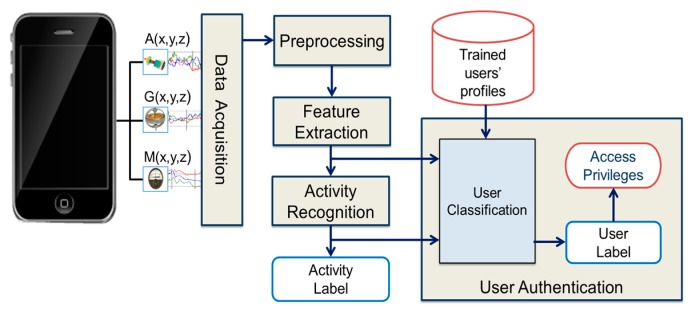
Proposed methodology for smartphone user authentication.

The proposed methodology for smartphone user authentication consists of five steps: data acquisition, data pre-processing, feature extraction, activity recognition, and user authentication. Figure 2 shows the proposed methodology with different steps. The detailed explanation related to each step is explained in the following sections.

### 4.1. Data Acquisition

The implementation of the proposed scheme for smartphone user authentication is based on a supervised machine learning approach. For carrying out experiments according to the proposed scheme, an existing dataset for physical activity recognition [20,21] was used. The data of 10 participants were collected for six different physical activities: walking, sitting, standing, running, walking upstairs, and walking downstairs. During data collection experiments, all participants performed every activity for three minutes. All participants were male, aged between 25 and 30. The experiments for data collection were conducted in one of the university buildings. For the purpose of walking and running activities, the corridor of a department was used, whereas for sitting and standing activities, university offices were used. A five-floor building was used for walking upstairs and downstairs activities. Every participant was equipped with five Samsung Galaxy S-II (i9100) smartphones at five different positions, including left and right jeans pockets, right upper arm, right wrist, and the waist position near the right leg. The smartphones were kept in portrait orientation for all body positions except the waist position, where the smartphone was held in a landscape orientation using a belt clip. The data were collected at a rate of 50 Hz from the smartphone inertial sensors. This sampling rate was selected to efficiently distinguish human physical activities in the later part of the experiment. Three sensors’ data were extracted from the dataset for this study, including an accelerometer, a gyroscope, and a magnetometer. The data collected from these sensors was in the form $\left\{a_{x},a_{y},a_{z},g_{x},g_{y},g_{z},b_{x},b_{y},b_{z}\right\}\in {\mathbb{R}}^{9}$, where ‘a’ represents the acceleration in meters per second square ($m/s^{2}$), ‘g’ represent the angular rotation measured by the gyroscope in radians per second (rad/s), and ‘b’ represents the magnet field strength measured by the magnetometer in micro tesla (µT), along the *x*-axis, *y*-axis, and *z*-axis.

### 4.2. Data Pre-Processing

The data recorded from the smartphone inertial sensors include unwanted noise generated from the participants and the sensors themselves. It was essential to eliminate the unwanted noise from the data before any further processing. Data pre-processing was employed to mitigate the unwanted noise from the sensors data and divide the data into small segments for better feature extraction. Data pre-processing was done in two steps.

#### 4.2.1. Noise Removal

For the purpose of noise removal, an average smoothing filter discussed in [25] was applied on each data sample value along every axis. The filter replaced each raw data sample value by the average value of its two adjacent data samples to reduce noise such as an abrupt spike that might have been generated because of phone suddenly falling to the ground. The average smoothing filter also eliminated the noise generated because of the vibrant motion of the participants during data recording.

#### 4.2.2. Data Segmentation

The orientation sensitivity of the smartphone inertial sensors influences the performance of activity recognition algorithms because the readings of these sensors are influenced by changing the orientation of the smartphone [58]. Most related studies assumed a fixed orientation of the smartphone while assessing different classification algorithms [59]. To address the issue of orientation sensitivity, a fourth dimension, i.e., magnitude of the sensor, was added to the existing three dimensions of each sensor. This was done because of the fact that the magnitude is not sensitive to the orientation. The magnitude of the sensor was calculated as given in Equation (1):

(1) $$mag=\sqrt{x^{2}+y^{2}+z^{2}},$$

where x, y, and z represent the *x*-axis, *y*-axis, and *z*-axis, respectively.

After adding magnitude, each sensor’s data was comprised of four dimensions: (x, y, z, mag). For better feature extraction and classifier training, it was necessary to divide the sensor data along each axis into small segments. A fixed-size window segmentation scheme was employed for this purpose because of its low computational complexity and most common use in activity recognition algorithms [25]. The size of the segmentation window was an important issue to analyze during data segmentation as the final accuracy of recognition was reliant on the window size. For this purpose, existing studies on physical activity recognition were analyzed, which showed that a time interval of nearly 5 *s* is sufficient to identify and recognize a physical activity [20,59]. Therefore, a fixed-width slicing window of 5 *s* in time (250 samples with 50 Hz sampling rate), with no overlap between the samples, was selected for dividing the raw data obtained from every sensor (along each axis) into small chunks of 5 *s*.

### 4.3. Feature Extraction

In any data mining scheme, it is critical to extract correct features for efficient recognition performance. This research places an emphasis on the recognition of six different physical activities performed by a user while keeping the smartphone at five different body positions. For this purpose, 12 different features were extracted from both time and frequency domains. Table 3 shows the set of features extracted for the recognition of activities of daily living selected in this study. These features have been selected because of their efficient performance in the state of the art for activity recognition using smartphone sensors. The existing studies [20,21,25,58,59,60] have discussed the excellent performance of these features in activity recognition experiments. A key reason of using more time domain features is their effective computational cost as compare to the frequency domain features. The frequency domain features are computationally complex and costly due to the expensive Fourier transformation [20,59] making them less feasible for our target smartphone platform.

### 4.4. Activity Recognition

From the perspective of data mining, activity recognition is considered a multi-class classification problem. Classifiers are machine learning algorithms that learn essential information from the features extracted from the signal, and then make classification decisions on the basis of these features [61]. In this work, prevalent classifiers that have been used in the state of the art for activity recognition were explored, and four different classifiers were used for this purpose so that an efficient comparison can be made of these classifiers’ performance in activity recognition. These classifiers include Decision Tree [38], K-Nearest Neighbors Classifier [46], Support Vector Machine [42], and Bayesian Network/Bayes Net Classifier [39]. For SVM classifier, a Sequential Minimal Optimization (SMO) [62] algorithm was used in this study.

### 4.5. User Authentication

The final step of the proposed methodology is user authentication, i.e., identifying and classifying a smartphone user as authenticated, supplementary, or impostor, and assigning a selected level of smartphone access privileges to that user. The user classification was performed on the basis of activity recognition, using a probabilistic scoring model. After user classification, zero-level access privileges were assigned to an impostor, i.e., no data access rights were given to an impostor user at all. A restricted-level of smartphone access was provided to a supplementary user, whereas full-level access rights to smartphone data and information were given to the authenticated smartphone user. The following section provides a detailed explanation of the probabilistic scoring model employed for user classification.

#### 4.5.1. Probabilistic Scoring Model for User Classification

##### Activity Weighting

The probabilistic scoring model classified a smartphone user on the basis of the activity recognized after activity classification. All activities were detected and recognized with different individual accuracies. This might have influenced the performance of user classification because an activity with lower recognition accuracy could have classified a user incorrectly. To avoid this issue, a weight $W_{\mathrm{activity}}$ was assigned to each of six selected activities according to their individual classification accuracies, e.g., $W_{\mathrm{walking}}$ was the weight assigned to the walking activity. An activity detected with higher classification accuracy was assigned a higher weight as compare to an activity that was detected with lower classification accuracy. The weight assigned to an activity was calculated by dividing its recognition accuracy with the overall average accuracy value of all activities, as given in Equation (2):

(2) $$W_{A_{r}}=\frac{Accuracy_{A_{r}}}{\frac{1}{M}\sum_{r=1}^{M}Accuracy_{A_{r}}},$$

where $A_{r}$ represents an activity label such as walking, running, sitting etc., and M is the total number of activities.

This weight value was used afterwards for calculating the classification score, as given in Equation (14), to classify a smartphone user as authenticated, supplementary, or impostor.

##### Computation of Trained Feature Vectors for Different User Classes

For the purpose of activity recognition and user classification, the feature vectors were computed by concatenating twelve different features extracted along all four dimensions, i.e., (*x*, *y*, *z*, mag), of accelerometer, gyroscope, and magnetometer. Each feature vector was of length 12 × 4 × 3 = 144, and computed over a data segment of 5 *s* (250 samples at a rate of 50 Hz) in time. Each activity’s data were collected for 3 min (180 *s*) duration for all body positions separately; therefore, 180/5=36 feature vectors were computed corresponding to each activity for a single body position. Overall, 36 × 5 = 180 feature vectors were computed related to each activity for an individual user.

For each user class, six activities were performed by a random number of participants belonging to that specific user class. The user authentication model was trained separately corresponding to all these activities for different user classes by using 70% data (selected at random) for training. For each activity, the model was trained for five different body positions. For this purpose, *K*-means clustering [63] was applied separately on the features vectors corresponding to five different body positions for each selected activity. As a result, the feature vectors were split into a different number of clusters by varying the value of *K*, and the cluster analysis [64] was performed on the basis of the average silhouette values to get an idea of how well-separated the resulting clusters are. The silhouette value for each point in the data is actually a measure of how similar that point is to the points in its own cluster as compared to the points in other clusters. It ranges from +1 to −1 in such a way that a value close to +1 indicates the points in the data that are distant from the neighboring clusters, whereas a value near −1 indicates the points that are possibly assigned to the wrong cluster. A silhouette value of 0 represents the points that are not distinctive in one cluster or another. The highest average silhouette value indicates that the clusters are well-separated. For cluster analysis, the silhouette value $S_{i}$ for the *i*th point in the data was calculated as given in Equation (3):

(3) $$S_{i}=\frac{\left(b_{i}-a_{i}\right)}{\max\left(a_{i},b_{i}\right)},$$

where $a_{i}$ is the average distance from the *i*th point to all other points in the same cluster, and $b_{i}$ is the minimum average distance from the *i*th point to the points in a different cluster, minimized over clusters.

The silhouette analysis was performed separately on the data corresponding to all selected activities for five different body positions. The data of all three user classes was considered in the analysis. Table 4 shows a comparison of the average silhouette values obtained for different values of *K* by clustering the activity patterns corresponding to five different body positions for all three user classes. Only the average silhouette values computed over all three user classes are provided for each activity. The highest average silhouette value obtained corresponding to each activity at a specific body position is represented in bold. It can be observed from the table that *K* = 3 provides the highest average silhouette value for all selected activities at all body positions, except the walking downstairs activity (at the left thigh position) for which the highest average silhouette value is obtained for *K* = 2. It means that *K* = 3 provides the best results in clustering the activity patterns of different class users. Therefore, using *K*-means clustering, the feature vectors for all selected activities corresponding to each body position were divided into three clusters by selecting *K* = 3. The centroid of each cluster was taken as a trained feature vector. In this way, 5 × 3 = 15 trained feature vectors were generated for a single activity. Thus, for all six activities, a total of 15 × 6 = 90 feature vectors were computed per user class.

The robustness of the method was tested by analyzing how the change in training data may affect the number of resulting clusters and the cluster centroids. For this purpose, 20 random training sets were obtained by selecting 70% data randomly from each user class corresponding to all selected activities. While selecting data for new training sets, all five body positions were considered for an activity. For each training set, the cluster analysis was performed on all activity patterns for different values of *K* and it was observed that *K* = 3 provided the best average silhouette value for all activities. It means that updating the training set did not influence the number of resulting clusters. Hence, the activity patterns were split into three different clusters using *K*-means clustering, and the new centroids were computed from the resulting clusters to find out how these centroids differ from the previously learned centroids. For this purpose, the newly computed centroids were compared with the corresponding learned centroids on the basis of Euclidean distance, and the minimum distance from the best-matching learned centroid was calculated as given in Equation (4):

(4) $$d_{m}=\underset{1\leq m\leq K}{\arg\;\min}\|\bar{C_{n}}-C_{m}\|,$$

where $\bar{C_{n}}$ and $C_{m}$ denote the *n*th new centroid and the *m*th learned centroid, respectively, and 1≤n≤K.

**Figure 3:**
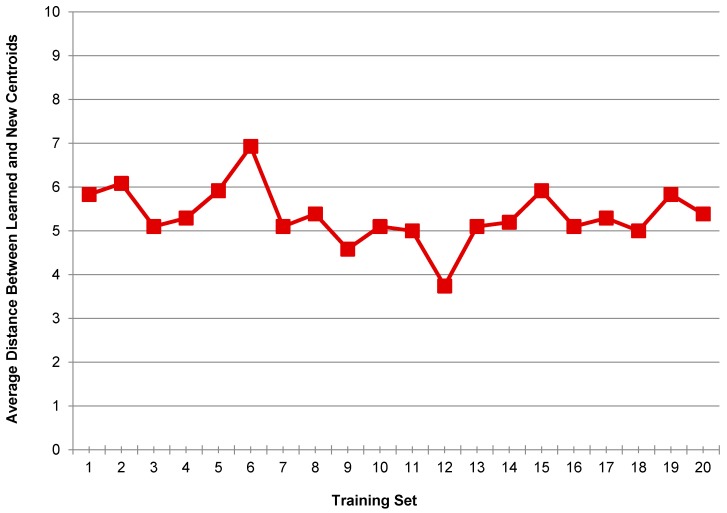
Average distance between the learned centroids and the new centroids for different training sets.

Finally, the average distance was calculated between the new centroids and the previously learned centroids by taking the mean value of all minimum distances computed for a training set. The mean distance values were calculated separately for all 20 training sets, which are plotted in Figure 3.

It can be seen from Figure 3 that the newly computed centroids differ from the learned centroids in accordance with the change in training data. As the training sets were generated based on the random data taken from each user class, hence these training sets were comprised of different data as compare to each other. This difference in the data was because different users had their own style of performing an activity, which gave rise to dissimilar activity patterns for different users.

On the basis of the results discussed above, it can be said that if new training data are added to the training set, it will not affect the number of clusters obtained by splitting different activity patterns using *K*-means clustering. So, the value of *K* will remain equal to 3. However, the cluster centroids will change according to the change in training data. Therefore, when new data are added to the training set in real time, the system updates the learned centroids according to the new training data. The updated centroids are then used as the trained feature vectors.

##### Calculation of Euclidean Distance between Feature Vectors

For testing of the user authentication model, the feature vectors were computed by extracting selected features from the rest of 30% data that was not used in training. Each testing feature vector was passed as an input to the activity recognition module for recognizing the activity performed by the user. Machine learning algorithms were used for the purpose of activity classification. After activity classification, the label of the activity recognized and its feature vector extracted from testing data were passed as inputs to the user authentication model for identifying the user type. For this purpose, the feature vector of the recognized activity was compared with the trained feature vectors, and Euclidean distance was computed between the feature vectors. As the label of recognized activity was known by the user authentication model, the extracted feature vector was compared only with the trained feature vectors corresponding to the recognized activity for all user classes. Hence, it saved computational time required for the comparison of the extracted feature vector with the trained feature vectors of all other activities.

Let $A_{r}$ represents the label of the activity recognized by the user authentication model, e.g., walking, standing, sitting, running, walking upstairs, or walking downstairs. The symbols $U_{auth}$, $U_{supp}$, and $U_{imp}$ represent smartphone users belonging to the authenticated, supplementary and impostor classes, respectively. Let $f_{A_{r}}$ represent the feature vector of the activity recognized from testing data, whereas $f_{A_{r},U_{auth}}$, $f_{A_{r},U_{supp}}$, and $f_{A_{r},U_{imp}}$ represent the trained feature vectors for the recognized activity corresponding to the authenticated, supplementary, and impostor classes, respectively. Euclidean distance [65] was calculated between these feature vectors using Equation (5):

(5) $$d\left(p,q\right)=\sqrt{\sum_{i=1}^{m}{\left(p_{i}-q_{i}\right)}^{2}},$$

where p and q represent two different feature vectors and *m* is the length of each feature vector.

Euclidean distance was computed between three different pairs of feature vectors as follows:

- $d\left(f_{A_{r}},f_{A_{r},U_{auth}}\right)$ represents Euclidean distance computed between the feature vector of the activity recognized and its trained feature vector for the authenticated user class.
- $d\left(f_{A_{r}},f_{A_{r},U_{supp}}\right)$ denotes Euclidean distance computed between the feature vector of the activity recognized and its trained feature vector for the supplementary user class.
- $d\left(f_{A_{r}},f_{A_{r},U_{imp}}\right)$ indicates Euclidean distance computed between the feature vector of the activity recognized and its trained feature vector for the impostor user class.

These distances were added together to find out the total distance $d_{total}$ as given in Equation (6):

(6) $$d_{total}=d\left(f_{A_{r}},f_{A_{r},U_{auth}}\right)+d\left(f_{A_{r}},f_{A_{r},U_{supp}}\right)+d\left(f_{A_{r}},f_{A_{r},U_{imp}}\right).$$

##### Calculation of Conditional Probabilities for Detecting Different Class Users

Euclidean distance computed between the feature vectors was used to find out the conditional probabilities of detecting a user as authenticated, supplementary, or impostor. These probabilities were calculated as follows:

(7) $$P\left(U_{auth}{|A}_{r}\right)=\frac{d_{total}-d\left(f_{A_{r}},f_{A_{r},U_{auth}}\right)}{2\;.d_{total}}$$

(8) $$P\left(U_{supp}{|A}_{r}\right)=\frac{d_{total}-d\left(f_{A_{r}},f_{A_{r},U_{supp}}\right)}{2\;.d_{total}}$$

(9) $$P\left(U_{imp}{|A}_{r}\right)=\frac{d_{total}-d\left(f_{A_{r}},f_{A_{r},U_{imp}}\right)}{2\;.d_{total}},$$

where $P\left(U_{auth}{|A}_{r}\right)$, $P\left(U_{supp}{|A}_{r}\right)$ and $P\left(U_{imp}{|A}_{r}\right)$ represent the conditional probabilities of detecting a user as authenticated, supplementary, or impostor respectively, given the activity recognized $A_{r}$.

These probability values were ranging from 0 to 1 and calculated in such a way that their sum was equal to 1, as shown in Equation (10):

(10) $$P\left(U_{auth}{|A}_{r}\right)+P\left(U_{supp}{|A}_{r}\right)+P\left(U_{imp}{|A}_{r}\right)=1.$$

To satisfy Equation (10), it is necessary that one of these probabilities should have a minimum value of 0.34. If all these probabilities are less than 0.34, then their summation can never be equal to 1. For this reason, the least maximum conditional probability value for a user class was taken as $P_{L_{max}}=0.34$.

##### Normalization of Conditional Probabilities

The conditional probability values of detecting different class users were scaled from their initial range, i.e., $\left[P_{min}\;P_{max}\right]=\left[01\right]$, to a new range, i.e., [$R_{min}\;R_{max}$], using Equation (11):

(11) $$P_{N}=\left(P-P_{min}\right).\frac{\left(R_{max}-R_{min}\right)}{P_{max}-P_{min}}+(R_{min}),$$

where P represents a probability value from 0 to 1, $P_{N}$ represents the normalized probability value of *P*, $P_{min}$ gives the minimum possible value of P that is equal to 0, $P_{max}$ denotes the maximum possible value of P that is equal to 1, $R_{min}$ represents the minimum value of $P_{N}$, and $R_{max}$ denotes the maximum value of $P_{N}$ that is kept equal to $P_{max}$.

The value of $R_{min}$ should be greater than or equal to the least maximum conditional probability value for any user class, i.e., $R_{min}\geq P_{L_{max}}$. If the conditional probability value for any user class is less than $P_{L_{max}}$, it means that one of the other two user classes has a maximum value of the conditional probability. Consequently, all conditional probability values less than $P_{L_{max}}$, i.e., 0.34, need to be discarded. For this reason, the probability values were normalized to a new range, i.e., [$R_{min}\;R_{max}$], such that, $P_{L_{max}}\leq R_{min}<1$ and $R_{max}=1$. Another purpose of normalizing these values to a higher range was to expand the classification score to a wider range for the efficient computation of the threshold values for classifying a user.

Let $P_{N}\left(U_{auth}{|A}_{r}\right)$, $P_{N}\left(U_{supp}{|A}_{r}\right)$ and $P_{N}\left(U_{imp}{|A}_{r}\right)$ represent the normalized conditional probabilities of detecting a user as authenticated, supplementary, or impostor, respectively. The range of these probability values was equal to $\left[R_{min\;}R_{max}\right]$. The maximum normalized probability $P_{N_{max}}$ was calculated using Equation (12):

(12) $$P_{N_{max}}=\max\{P_{N}\left(U_{auth}{|A}_{r}\right),P_{N}\left(U_{supp}{|A}_{r}\right),P_{N}\left(U_{imp}{|A}_{r}\right)\}.$$

##### Computation of Access Level Values for Multiple User Classes

An access level value $L_{A_{user}}$ was used for each user class, which represented the level of access privileges assigned to a user class. Generally, a higher value of the access level for a user class means that a user belonging to that specific class is allowed to access more data and information as compare to the users belonging to a user class with lower access level value. Therefore, the access level value was assigned to each user class in such a way that the authenticated user class had the maximum value, the impostor user class had the minimum value, and the supplementary user class had a median value for this access level, i.e., $L_{A_{auth}}>L_{A_{supp}}>L_{A_{imp}}$. This access level value was calculated on the basis of $R_{min}$ using Equation (13):

(13) $$L_{A_{user}}={\left(R_{min}\right)}^{n},$$

where *n* represents an integer that was assigned a value of 0, 1, or 2 depending upon the value of maximum normalized probability $P_{N_{max}}$.

The values were assigned to $L_{A_{user}}$ according to the following criteria:

- For $P_{N_{max}}=P_{N}\left(U_{auth}{|A}_{r}\right)$, the integer n was assigned a value of 0, i.e., n=0.
  Hence, from Equation (13), $L_{A_{user}}=L_{A_{auth}}={\left(R_{min}\right)}^{0}=1$.
- For $P_{N_{max}}=P_{N}\left(U_{supp}{|A}_{r}\right)$, the integer n was assigned a value of 1, i.e., n=1.
  Hence, from Equation (13), $L_{A_{user}}=L_{A_{supp}}={\left(R_{min}\right)}^{1}=R_{min}$.
- For $P_{N_{max}}=P_{N}\left(U_{imp}{|A}_{r}\right)$, the integer n was assigned a value of 2, i.e., n=2.
  Hence, from Equation (13), $L_{A_{user}}=L_{A_{imp}}={\left(R_{min}\right)}^{2}$.

##### Calculation of Classification Score

The classification score was calculated on the basis of the access level value assigned to a user class, weight of the activity recognized $W_{A_{r}}$ and the maximum normalized probability $P_{N_{max}}$, as given in Equation (14):

(14) $$c_{s}=L_{A_{user}}.W_{A_{r}}.P_{N_{max}},$$

where $A_{r}$ represents the label of the activity recognized, e.g., walking.

The classification score was scaled to a different range of values, depending upon the value of maximum normalized probability, according to the following criteria:

- For $P_{N_{max}}=P_{N}\left(U_{auth}{|A}_{r}\right)$, $L_{A_{user}}=L_{A_{auth}}=1$.
  Hence, from Equation (14),
  (15) $$c_{s}=W_{A_{r}}.P_{N_{max}}.$$
- For $P_{N_{max}}=P_{N}\left(U_{supp}{|A}_{r}\right)$, $L_{A_{user}}=L_{A_{supp}}=R_{min}$.
  Hence, from Equation (14),
  (16) $$c_{s}=R_{min}.W_{A_{r}}.P_{N_{max}}.$$
- For $P_{N_{max}}=P_{N}\left(U_{imp}{|A}_{r}\right)$, $L_{A_{user}}=L_{A_{imp}}={\left(R_{min}\right)}^{2}$ Hence, from Equation (14),
  (17) $$c_{s}={\left(R_{min}\right)}^{2}.W_{A_{r}}.P_{N_{max}}.$$

It can be observed from Equations (15) to (17) that even for the same value of the normalized probability across different user classes, the classification score will be different. If the weight of the activity recognized $W_{A_{r}}$ is considered as close to 1, and a median value of 0.67 is chosen for $R_{\min}$, then the classification score will have a different range of values depending upon the value of maximum normalized probability, as given below:

- If $P_{N_{max}}=P_{N}\left(U_{auth}{|A}_{r}\right)$, then $c_{s}$ will have a range near to [0.67 1], with $P_{N}\left(U_{auth}{|A}_{r}\right)$ having a range [0.67 1].
- If $P_{N_{max}}=P_{N}\left(U_{supp}{|A}_{r}\right)$, then $c_{s}$ will have a range near to [0.45 0.67], with $P_{N}\left(U_{supp}{|A}_{r}\right)$ having a range [0.67 1].
- If $P_{N_{max}}=P_{N}\left(U_{imp}{|A}_{r}\right)$, then $c_{s}$ will nearly have a range of values less than 0.45 with $P_{N}\left(U_{imp}{|A}_{r}\right)$ having a range of [0.67 1].

###### Calculation of Threshold Values for Classifying a Smartphone User

Two threshold values, i.e., ${Ʈ}_{1}$ and ${Ʈ}_{2}$, were used for classifying a smartphone user into one of three different user classes, such that ${Ʈ}_{1}<{Ʈ}_{2}$. These threshold values were calculated as given in Equations (18) and (19):

(18) $${Ʈ}_{1}=R_{min}.\left(P_{L_{max}}.(1-R_{min}\right)+(R_{min}))$$

(19) $${Ʈ}_{2}=\left(P_{L_{max}}.(1-R_{min}\right)+(R_{min})),$$

where $R_{min}$ represents the minimum possible value of the normalized conditional probability for a user class, and $P_{L_{max}}$ is the least maximum conditional probability value equal to 0.34.

These threshold values discarded the normalized probability values that were obtained corresponding to the conditional probability values less than $P_{L_{max}}$ because these values had no effect on user classification. The criteria used for classifying a smartphone user on the basis of classification score $c_{s}$ and threshold values ${Ʈ}_{1}$ and ${Ʈ}_{2}$ are as follows:

- For $0\leq c_{s}\leq {Ʈ}_{1}$, the user was classified as impostor.
- For ${Ʈ}_{1}<c_{s}\leq {Ʈ}_{2}$, the user was classified as supplementary.
- For $c_{s}>{Ʈ}_{2}$, the user was classified as authenticated.

####### Effect of Varying $R_{min}$ on Threshold Values and User Classification

It can be seen from Equations (14) to (19) that the classification score and the threshold values are dependent on the value of $R_{min}$. Any change in the value of $R_{min}$ will result in a change in the classification score. The threshold values are computed in such a way that upon any change in the value of $R_{min}$, these values will get updated according to the new classification score to avoid any significant change in the user classification results. While testing user classification, a median value, i.e., 0.67, was selected for $R_{min}$, considering that $0.34\leq R_{min}<1$. The classification score and the threshold values were then computed accordingly.

**Figure 4:**
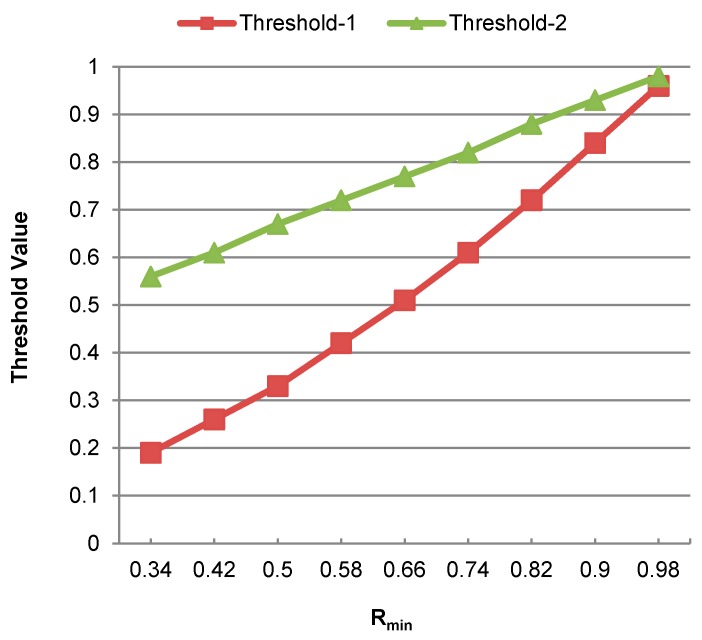
Effect of varying $R_{min}$ value on the threshold values ${Ʈ}_{1}$ and ${Ʈ}_{2}$.

Figure 4 shows the effect of varying $R_{min}$ on the threshold values ${Ʈ}_{1}$ and ${Ʈ}_{2}$, which are represented by Threshold-1 and Threshold-2, respectively. It can be observed that the difference between these threshold values, i.e., ${Ʈ}_{1}$ and ${Ʈ}_{2}$, is decreased by increasing the value of $R_{min}$. If a maximum value is taken for $R_{min}$, the difference between these threshold values becomes least. Conversely, if $R_{min}$ is assigned a minimum value of 0.34, then the difference between these threshold values becomes maximum. In both these cases, the results of user classification may not be proficient because the margin between these threshold values and the classification score will either become too small or too large, which may influence the user classification results. Hence, a median value of $R_{min}$ is more suitable for efficient user classification.

## 5. Results and Performance Analysis

For the purpose of smartphone user authentication, the proposed framework utilized activity recognition and user classification. The user classification was performed by means of activity recognition. To evaluate the performance of the proposed scheme, the experimental results are presented in two different sections separately for activity recognition and user classification. Following sections discuss these results.

### 5.1. Performance Analysis of Activity Recognition

In order to evaluate the performance of activity recognition for this study, four different classification algorithms including Decision Tree (DT), Bayes Net (BN), K-Nearest Neighbor (K-NN), and Support Vector Machine (SVM) were trained and evaluated on the selected dataset. These classifiers were selected because they have been used in the state of the art for activity recognition [20,21,25,59]. To ensure fairness in activity recognition results, a 10-fold stratified cross validation scheme was used for evaluating the performance of these classifiers. Hence, all activity traces in the dataset were split randomly into 10 sets, and iterated 10 times in such a way that every set of data was selected to use for testing and remaining sets were employed for training of the classifiers. Only the average results of all 10 repetitions are included in this section. The performance metrics used in this study for evaluating the classifiers performance for activity recognition are computational time taken, average accuracy rate, f-measure, kappa statistic, Root Mean Squared Error (RMSE) and Mean Absolute Error (MAE). The kappa statistic is a measure that is independent of the total number of classes and the number of samples per class. A kappa statistic value of *k* = 0 represents a chance level classification performance, whereas in case of perfect classification, *k* reaches its best value of 1. If *k* < 0, it means that the performance of classification is poorer than the chance level classification performance. These performance metrics are evaluated for all four classifiers selected for activity recognition, and the results of activity classification are computed separately for all five body positions selected in this study.

**Figure 5:**
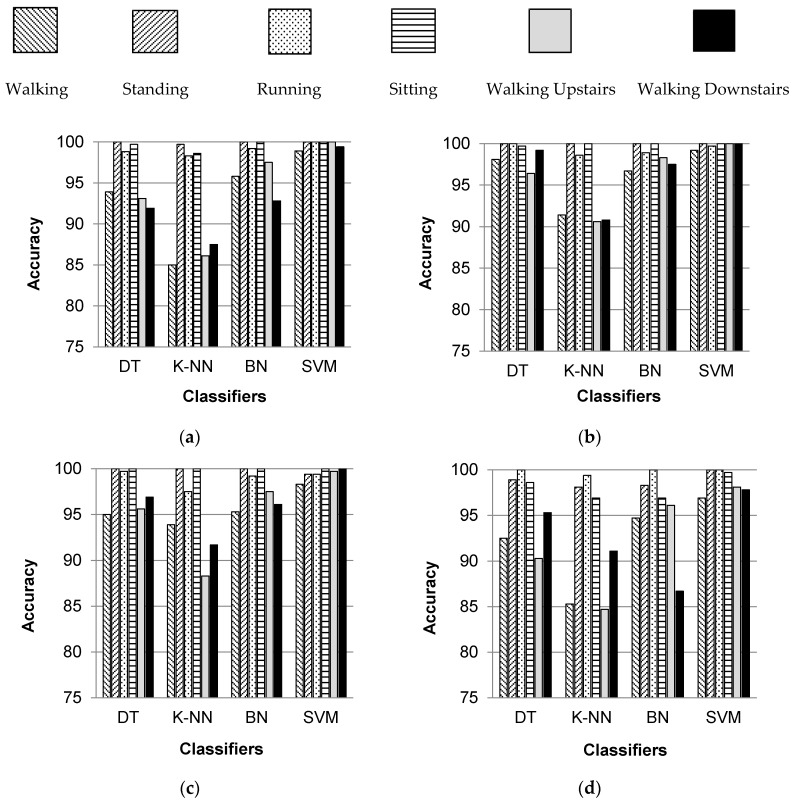

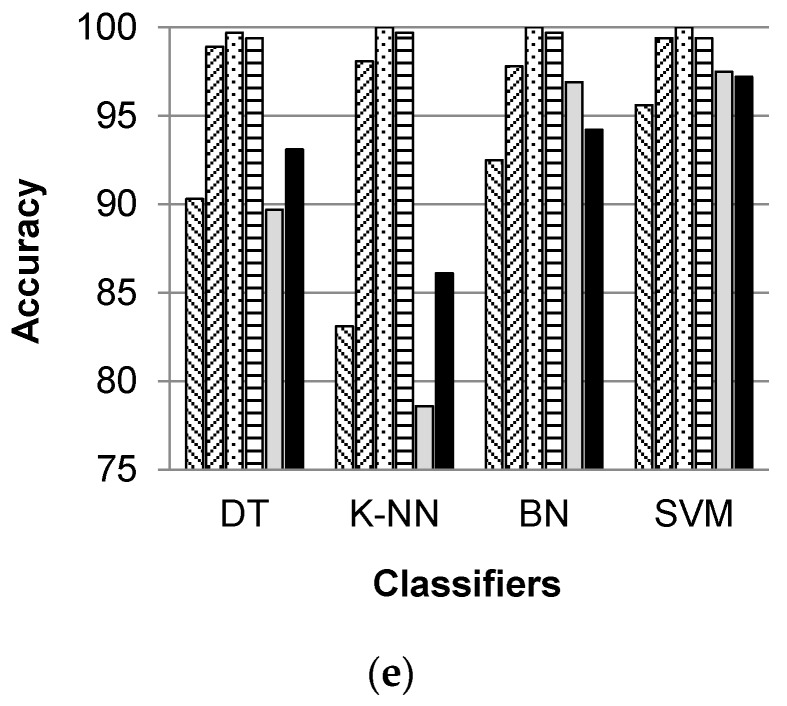
Individual classification accuracies of selected activities when classified with DT, K-NN, BN, and SVM classifiers for five different body positions: (**a**) waist; (**b**) left thigh; (**c**) right thigh; (**d**) upper arm; (**e**) wrist.

Figure 5 shows the individual percentage accuracies of classification for all selected activities over five body positions when classified with DT, K-NN, BN and SVM classifiers. It can be observed that the individual classification accuracies of standing, running, and sitting activities are higher irrespective of the classifier and the body position. Thus, it can be said that these activities are more easily recognizable than other selected activities. The activities of sitting and standing are distinguished from each other on the basis of the fact that the orientation of the smartphone placed on a human body changes when a user changes his/her posture or stance for sitting and standing. Thus, the readings of smartphone inertial sensors are different. The classification of walking, walking upstairs, and walking downstairs activities is position dependent, and gives better results if the smartphone is placed in the left or right jeans pocket. All six activities are recognized with higher individual accuracies when classified with SVM and BN classifiers.

Table 5 shows the performance parameters of the selected classifiers for activity recognition at five different body positions. It can easily be observed that SVM classifier provides the best average accuracy rate as compare to the accuracy rate values provided by DT, K-NN, and BN classifiers. On the other hand, the error rate for SVM classifier, evaluated by MAE and RMSE, is also very high for all body positions. Table 6 shows the average values of individual performance metrics for all selected classifiers. It can be seen that the overall average values of accuracy rate, kappa statistic, and f-measure are higher and comparable for SVM, BN, and DT classifiers. However, K-NN provides lower accuracy rate along with lower kappa statistic and f-measure values. The average accuracy rate for SVM classifier is 99.18%, which is 2.36%, 1.8%, and 5.88% higher than the average accuracy rate of DT, BN, and K-NN classifier, respectively. The values of MAE and RMSE for SVM classifier are 0.22 and 0.312 respectively, which are higher as compare to the error rate values for DT and BN classifiers. The average accuracy rate of BN classifier is 0.56% and 4.08% higher than the average accuracy rate of DT and K-NN classifier, respectively. Also, the error rate of BN classifier is better than the error rates provided by DT, SVM, and K-NN classifiers.

**Figure 6:**
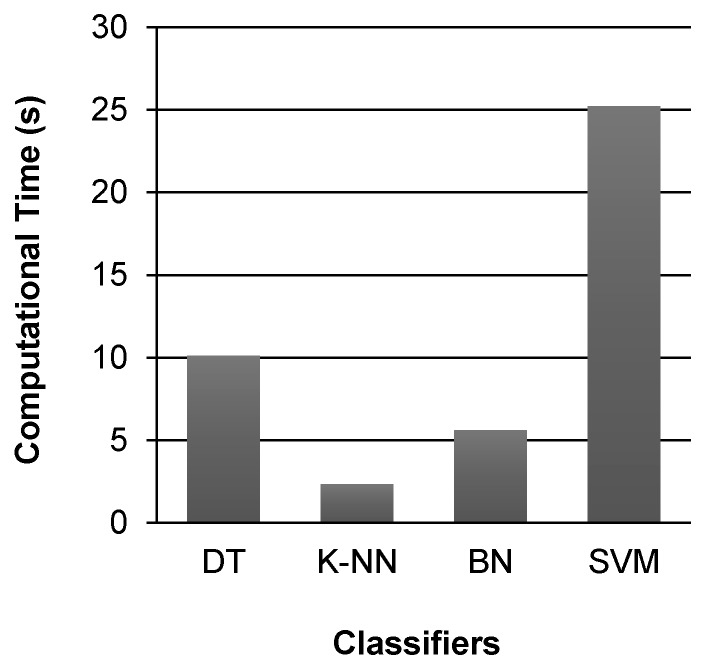
Computational time taken by different classifiers for activity classification.

Another important performance metric for evaluating the performance of these classifiers is their computational complexity, which effects the time taken by each classifier for building training model and performing classification. Figure 6 shows a comparison of the computational time taken by the selected classifiers for activity classification. It can be observed that K-NN classifier takes less time as compare to all other classifiers. The time taken by SVM classifier for activity classification is 25.21 s, which is 10.8 times more than the time (2.32 s) taken by K-NN classifier, and 4.5 times more than the time (5.61 s) taken by BN classifier to perform classification. The time taken by DT classifier for activity classification is 10.11 s.

On the basis of the results discussed above, it can be said that the overall performance of Bayes Net classifier in classifying the selected activities is better than other classifiers performance. Although, SVM provides the best accuracy rate for activity classification, but its error magnitude is also quite higher. On the other hand, the BN classifier provides an accuracy rate that is comparable to the accuracy rate of SVM classifier, but its error rate is very small. Also, the SVM classifier is computationally expensive, and it takes significantly more time for building a training model and performing classification. As a smartphone is equipped with limited processing power, memory, and storage, therefore, it is not feasible to use SVM classifier for on-device activity classification in real-time. Otherwise, the battery power will be drained quickly, and the output will be delayed because of the extensive computational time taken by SVM classifier for classification. Bayes Net classifier is based on a probabilistic model that is computationally very simple [39]. Hence, it takes less time in building and updating the training model, and performing on-device activity classification in real time. This suggests the Bayes Net classifier as an optimal choice for online activity recognition using smartphone sensors.

### 5.2. Performance Analysis of User Classification

The user authentication was done by means of user classification based on activity recognition. For user classification, three user classes were considered, including authenticated, supplementary, and impostor class. The dataset used for the activity recognition was pre-labeled for all activities performed by 10 participants/users. However, there were no user class labels for the participants in the dataset. Our idea was to utilize the dataset for learning the activity patterns of different users or a set of users. For this reason, the users in the dataset were randomly divided into three folds, i.e., Fold-1, Fold-2, and Fold-3. Fold-1 and Fold-2 represented the sets of users belonging to the authenticated and supplementary classes, respectively, whereas Fold-3 contained the set of users belonging to the impostor class. Five different scenarios were taken for the distribution of 10 users among these folds, as shown in Table 7. For each scenario, all possible permutations of the users were applied on three folds iteratively in such a way that every user became a part of each fold at least once.

For validating the user classification results, a 70%-30% split was used for training and testing data, respectively. For this purpose, each fold of data representing a specific user class was randomly partitioned into two independent sets, i.e., training and testing sets, where 70% of the data were selected for training the user classification model, and the remaining 30% were used for testing. The authors in [66] performed a broad simulation study for the purpose of evaluating commonly used splitting strategies for testing and training data, which concluded that allocating two-thirds (nearly 67%) of the data for training provides better classification accuracy. Moreover, the existing studies [60,67] also utilized a 70%/30% split for training and testing data, respectively, which provided efficient results for physical activity recognition. For every user class, the authentication model was trained to recognize six selected activities performed by the user while carrying the smartphone at five different body positions. The research work in [20,21,58,59] showed that a time interval of 4–5 *s* is sufficient for the recognition of a physical activity, considering a sampling rate of 50 Hz. For this purpose, the user authentication model was trained to identify a user with the activity duration of 5 *s*. During testing of user classification, the selected features were extracted from testing data over a data segment of 5 *s* in time, having 250 samples at a rate 50 Hz. The activity performed by the user was recognized based on these extracted features. After that, the recognized activity and the extracted features were passed to the user authentication model. The probabilistic scoring model was applied on the extracted features to calculate classification score on the basis of Euclidean distance between different feature vectors, using Equation (14). A median value of 0.67 was taken for $R_{min}$ initially and the threshold values ${Ʈ}_{1}$ and ${Ʈ}_{2}$ were calculated using Equations (18) and (19). The user was classified as authenticated, supplementary, or impostor based on these threshold values.

**Figure 7:**
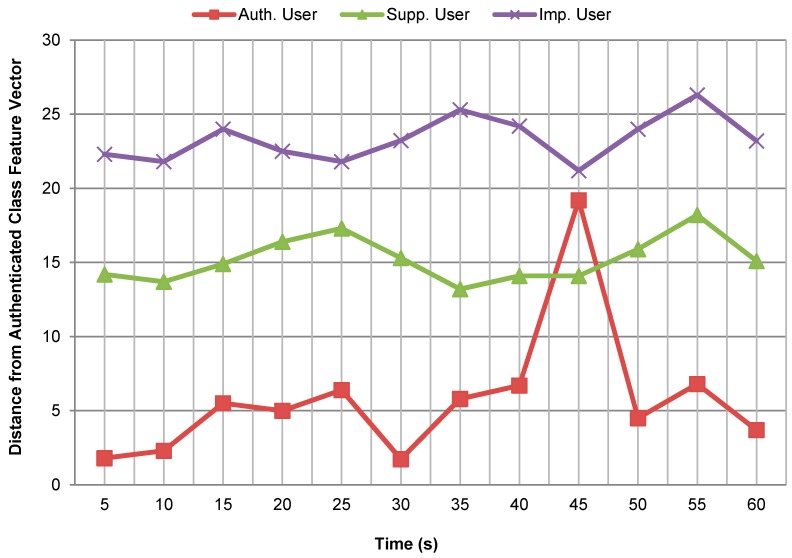
Euclidean distance between the authenticated user class feature vector and the feature vectors computed from testing data for different candidate users.

Figure 7 shows Euclidean distance between the trained feature vector for the authenticated class and the feature vectors computed from testing data for different class users to illustrate how sure the system is about the authentication. The trained feature vector for the authenticated class was selected corresponding to the walking activity for the left thigh position in this case. Similarly, the feature vectors for the different candidate users were also computed corresponding to the walking activity for the left thigh position over the data segments of 5 *s*. The distance was calculated after every 5 s’ interval of time for the activity duration of one minute only. From Figure 7, it can be observed that the distance of the authenticated class feature vector from the authenticated user is very small for all time intervals (except at interval from 41 s to 45 s) as compare to its distance from the supplementary and imposter users. Also, the distance values computed at the same time for the different candidate users have a wide gap for most of the time intervals. It can be observed that both supplementary and impostor users are well separated from the authenticated user on the basis of the computed distances. Also, the supplementary and impostor users are at a fair distance from each other in this case. This shows that the system is quite sure about the authenticity of different class users for most of the time intervals.

**Figure 8:**
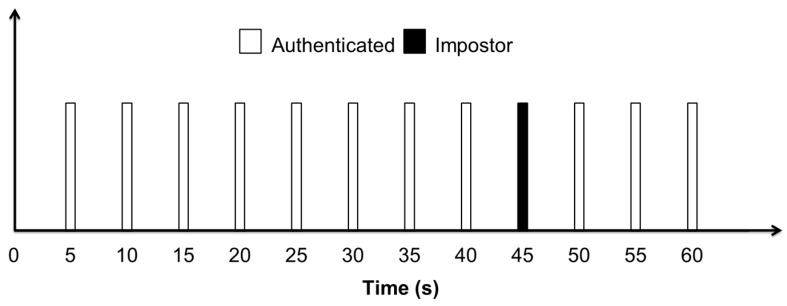
Output of the classification model at different time intervals while classifying a candidate user belonging to the authenticated class.

Looking at the relative distances of the different candidate users from the authenticated class only (as shown in Figure 7), the output of the system cannot be realized. It is necessary to compute the distance of each candidate user from all user classes in order to know the output of the system. For example, to find the output of the system while classifying the authenticated user (whose distance is plotted from the authenticated class in Figure 7), the distance of this specific user was calculated from other user classes as well. From Figure 7, it can be observed that the distance of this particular user from the authenticated class is very large for the time interval from . Therefore, after this time interval the user was misclassified as an imposter because the user had minimum distance from the imposter class. Figure 8 shows the output of the system after every five seconds’ interval of time while classifying this candidate user. It can be seen that the system has correctly recognized the user as an authenticated user for all time intervals, except the time interval from 41 s to 45 s, after which the user was classified as an impostor. So, after a period of one minute, it can be said that the system has correctly identified that the smartphone was possessed by an authenticated person with a very high accuracy of 91.67%. These results also suggest that an activity pattern of 5 *s* duration is sufficient to recognize and classify a user, considering a sampling rate of 50 Hz.

The results of the user classification were computed iteratively for all possible permutations of the users across three folds, considering all the scenarios given in Table 7. Only the average results of all iterations are included in this section. To thoroughly analyze the results, different values were chosen for $R_{min}$ but no significant changes were observed in the results that were obtained corresponding to the initial value of $R_{min}=0.67$. Therefore, this section reports the average results of the user classification for only the initial value of $R_{min}$. The metrics used to evaluate the user classification performance are True Positive Rate (TPR), False Positive Rate (FPR), accuracy, precision, recall, and f-measure. Table 8 shows the results of user classification based on activity recognition at five different body positions. It can be seen that for all body positions, the value of TPR is higher for the impostor class, which means that the authentication model has identified the impostor users more accurately as compare to the authenticated and supplementary users.

**Figure 9:**
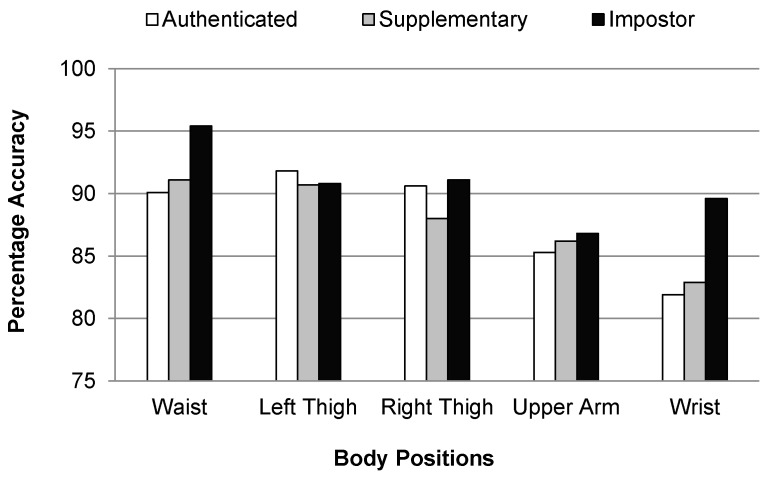
Individual classification accuracies of different user classes at five different body positions.

Figure 9 shows that the individual classification accuracies of all the user classes are higher for the waist, left thigh, and right thigh positions. It means that it is easy to identify a user by recognizing an activity if the smartphone is placed in the left or right jeans pocket, or hung from a belt clip at the waist. This is due to the fact that the performance of activity recognition is better for these body positions as compared with other body positions used, as can be seen in Figure 5. The overall classification accuracies for the authenticated, supplementary, and impostor classes are 87.94%, 87.78%, and 90.74%, respectively.

Generally, a smartphone has a single authenticated user and a few supplementary users only. All other users of the smartphone may be treated as impostors. Therefore, the impostor user class has a large number of instances as compare to the authenticated and supplementary user classes. For real-time authentication, the proposed framework requires the recording of training data for the authenticated and supplementary class users only. During training, the system extracts different features from the data recorded for the authenticated and supplementary class users, and divides the feature vectors computed corresponding to different activities into *K* clusters using *K*-means clustering. The system then takes the learned centroids as the trained feature vectors for authenticated and supplementary classes. If new training data are added to the training set, then the training data are clustered again using *K*-means clustering and the centroids are updated in accordance with the new data. After computing trained feature vectors, the system calculates the distance of each computed feature vector from the trained feature vectors and finds the maximum possible distance $d_{max}$. During real-time authentication, the system considers a smartphone user as an impostor by default until and unless a definite matching pattern is detected for an activity in the trained users’ profiles. It extracts the feature vector from the real-time data obtained from the smartphone sensors and recognizes the activity performed by the user. The extracted feature vector is then compared with the trained feature vectors of the authenticated and supplementary classes, and user classification is performed. In the case of an authenticated or a supplementary user, the extracted feature vector will be matched with one of the trained feature vectors and the Euclidean distance between the matched similar feature vectors will be less than or equal to $d_{max}$. Hence, the user will be classified as an authenticated or a supplementary user. On the other hand, if the user is an impostor, his/her activity pattern will be different from those of trained activity patterns. So, the Euclidean distance between the feature vector extracted for an impostor’s activity and that of trained feature vectors will be higher than $d_{max}$ and the user will be recognized as an impostor. In this way, the system ably handles a new smartphone user as an impostor whose activity pattern is not yet determined.

## 6. Conclusions

In this paper, smartphone user authentication based on physical activity recognition using mobile sensing has been analyzed. A novel multi-class user classification scheme is presented for the authentication of smartphone users, which is based on physical activity recognition incorporated with micro-environment sensing. Twelve different features from time and frequency domains are extracted for the recognition of six different activities, i.e., walking, standing, sitting, running, walking upstairs, and walking downstairs. The smartphone users are classified into three classes, i.e., authenticated, supplementary, and impostor, by recognizing their activity patterns using a probabilistic scoring model. It is observed that the recognition of standing, running, and sitting activities is easier irrespective of the smartphone position on the human body. As a result, it is easy to identify a smartphone user on the basis of these activities. On the other hand, the activities of walking, walking upstairs, and walking downstairs are smartphone position-dependent. These activities can be best recognized only if the smartphone is placed in the left or right jeans pocket, or hung with a belt clipper at the waist position. This shows that these positions are best suited for placing a smartphone on the human body for user authentication based on activity recognition. Moreover, it is noticed that the Bayes Net classifier provides the best performance for on-device activity recognition in terms of accuracy, error rate, and computational time required for activity classification. Hence, these findings conclude that the Bayes Net classifier is an optimal choice for online authentication of a smartphone user based on physical activity recognition.

This work can be extended to detect, recognize, and trace more complex activities for smartphone user authentication. For this purpose, more sensors can be added to the framework including virtual sensors such as apps usage, etc., to learn and recognize the behavior of a smartphone user while using smartphone. In case of a large amount of input data, the processing can be done on the cloud instead of the device itself. During real-time authentication of a smartphone user, because of noise and other interference, the data collected from the smartphone sensors may give rise to a random or abnormal activity sequence that can incorrectly classify a smartphone user. A knowledge-based authentication can be incorporated into the framework along with behavioral authentication to improve the accuracy of user classification. A list of security questions can be added to the framework. The answers to these questions should only be acknowledged by the authenticated user. If an abnormal activity pattern is detected by the system and the calculated classification score falls in a particular range, then a random security question can be asked. In case of a correct answer, the classification score can be updated by adding an additional score for the right answer. In case of a wrong answer, the classification score can be decreased. The updated classification score can then be compared with the threshold values for classifying the smartphone user. To further expand the work, the location of a smartphone user can be traced using smartphone sensors and his/her activity information can be acquired. This information can be further utilized for different purposes including forensic analysis and crime investigations.

## Figures and Tables

**Table 1:** A comparison of different studies of behavioral authentication of smartphone users.

Study	Behavioral Biometrics Approach	Classifier	Feature Set
Yang et al. [11], 2013	Hand waving using linear accelerometer	-	Sampling interval, acceleration along *x*, *y* and *z* axes
Shrestha et al. [41], 2015	Hand wavingusing ambient light sensor	SVM [42]	Timestamps, light intensity, hand wave gesture duration
Draffin et al. [8], 2014	Keystroke biometrics	Neural Network Classifier [43]	Location pressed on key, length of press, size of touched area, drift
Feng et al. [44], 2013	Keystroke biometrics	Decision Tree [38], Bayes Net [39] Random Forest [45],	-
Frank et al. [14], 2013	Touchscreen interactions	SVM [42], K-NN [46],	-
Shahzad et al. [47], 2012	Touchscreen interactions	-	-
Derawi et al. [48], 2010	Gait biometrics using smartphone sensors	DTW [49]	Time interpolation, Average cycle length
Mantyjarvi et al. [50], 2005	Gait biometrics using accelerometer	-	Acceleration along *x*, *y* and *z* axes, 10 bin FFT histograms
Clarke and Mekala et al. [51], 2007	Dynamic signatures by typing words	-	-
Sae-Bae [52], 2014	Line signature drawn with fingertip	DTW [49],	-
Kunz et al. [53], 2011	Speaker verification during ongoing phone call	HMMs [54]	-
Das et al. [55], 2008	Speaker’s identification based on speech dynamics	DTW [49]	-
Kambourakis et al. [56], 2014	Behavioral profiling	MLP [40], Random Forest [45], K-NN [46]	Hold time, inter-time, speed, distance

**Table 2:** Limitations of behavioral biometric approaches for smartphone user authentication.

Behavioral Biometric Approach	Limitations
Hand waving Patterns and Gestures	Requires a user to interact with the device actively and make a specific hand waving gesture for authentication User may generate some random hand waving gestures un-intentionally Validates a user only when a hand waving gesture is made Failure in identifying an impostor who accesses the phone while it is unlocked Multiple users may have the same hand waving patterns
Keystroke Dynamics	Requires active interaction of the user with the device keyboard for authentication Validates a user only when something is typed using the device keyboard Learning the keystroke patterns for a new user takes a lot of time Person’s typing behavior changes considerably throughout a day with different states of mind such as excited, tired, etc. Switching keyboards may change the typing patterns Disruptions during typing may significantly influence the typing patterns
Touchscreen Interactions	Requires active interaction of the user with the touchscreen for authentication Holding a smartphone in hands with different orientations vary the way of the user’s interactions with the touchscreen User’s activity while interacting with touchscreen, such as walking, sitting, standing etc., effects the way of touching the device screen
Handwriting and Signatures	Requires a user to interact with the device actively to input signatures Only feasible for entry point authentication People may not sign in a steady way all the times
Voice	Unwanted noises in the user’s surroundings, such as traffic noise, noise of a crowd of people talking etc., greatly affect the recognition and identification of the user’s voice
Gait Patterns	Wearing an outfit, such as a trench coat or a footwear, may change a person’s walking style Dependency of gait patterns on the position of motion sensors on the human body
Behavioral Profiling	User’s behavioral patterns change with the user’s mood and state of mind while interacting with different services and applications using touchscreen and keystroke

**Table 3:** A set of features extracted for activity recognition and user authentication.

Feature	Symbol	Formula	Domain
Max. Amplitude	$s_{\max}$	$s_{\max}=\max\left\{s\left(t\right)\right\}$	Time
Min. Amplitude	$s_{\min}$	$s_{\min}=\min\left\{s\left(t\right)\right\}$	Time
Mean	μ	$\mu =\frac{1}{N}\sum s\left(t\right)$	Time
Variance	$\sigma^{2}$	$\sigma^{2}=\frac{1}{N}\sum {\left(s\left(t\right)-\mu \right)}^{2}$	Time
Kurtosis	K	$K=m_{4}/m_{2}^{2}$	Time
Skewness	S	$S=(m_{3})/\left(m_{3}^{3/2}\right)$	Time
Peak-to-Peak Signal Value	$s_{\mathrm{pp}}$	$s_{\mathrm{pp}}=s_{\max}-s_{\min}$	Time
Peak-to-Peak Time	$t_{\mathrm{pp}}$	$t_{\mathrm{pp}}=t_{s_{\max}}+t_{s_{\min}}$ $t_{s_{\max}}=\left\{t|s\left(t\right)=s_{\max}\right\}$ $t_{s_{\min}}=\left\{t|s\left(t\right)=s_{\min}\right\}$	Time
Peak-to-Peak Slope	$s_{\mathrm{pps}}$	$s_{\mathrm{pps}}=s_{\mathrm{pp}}/t_{\mathrm{pp}}$	Time
Absolute Latency to Amplitude Ratio	ALAR	$\mathrm{ALAR}=\left|t_{s_{\max}}/s_{\max}\right|$	Time
Energy	$E_{f}$	$E_{f}=\sum {\left|S\left(f\right)\right|}^{2}$	Freq.
Entropy	H(S(f))	$H\left(S\left(f\right)\right)=-\overset{N}{\underset{i=1}{\sum }}p_{i}\left(S\left(f\right)\right)\log_{2}p_{i}\left(S\left(f\right)\right)$	Freq.

**Table 4:** Cluster analysis based on the average silhouette values for different values of *K*.

Activity	*K* = 2	*K* = 3	*K* = 4	*K* = 5	*K* = 6	Body Position
Walking	0.74	**0.75**	0.63	0.51	0.50	Waist
	0.81	**0.84**	0.76	0.57	0.56	Left Thigh
	0.71	**0.72**	0.67	0.58	0.49	Right Thigh
	0.79	**0.80**	0.73	0.63	0.60	Upper Arm
	0.80	**0.87**	0.65	0.51	0.40	Wrist
Sitting	0.64	**0.73**	0.58	0.46	0.43	Waist
	0.68	**0.70**	0.63	0.50	0.45	Left Thigh
	0.80	**0.84**	0.76	0.51	0.50	Right Thigh
	0.71	**0.79**	0.60	0.51	0.49	Upper Arm
	0.64	**0.71**	0.56	0.40	0.20	Wrist
Standing	0.61	**0.70**	0.53	0.41	0.43	Waist
	0.71	**0.80**	0.72	0.61	0.60	Left Thigh
	0.54	**0.75**	0.51	0.33	0.31	Right Thigh
	**0.61**	**0.61**	0.44	0.32	0.30	Upper Arm
	0.74	**0.80**	0.65	0.50	0.48	Wrist
Running	0.54	**0.60**	0.43	0.36	0.35	Waist
	0.79	**0.86**	0.76	0.57	0.50	Left Thigh
	0.51	**0.65**	0.41	0.21	0.21	Right Thigh
	0.46	**0.75**	0.62	0.41	0.41	Upper Arm
	0.84	**0.87**	0.70	0.50	0.49	Wrist
Sitting	0.64	**0.73**	0.58	0.46	0.43	Waist
	0.68	**0.70**	0.63	0.50	0.45	Left Thigh
	0.80	**0.84**	0.76	0.51	0.50	Right Thigh
	0.71	**0.79**	0.60	0.51	0.49	Upper Arm
	0.64	**0.71**	0.56	0.40	0.20	Wrist
Walking Upstairs	0.71	**0.79**	0.63	0.56	0.49	Waist
	**0.82**	**0.82**	0.73	0.54	0.50	Left Thigh
	0.77	**0.81**	0.70	0.61	0.60	Right Thigh
	0.70	**0.75**	0.51	0.44	0.40	Upper Arm
	0.51	**0.61**	0.46	0.25	0.24	Wrist
Walking Downstairs	0.81	**0.88**	0.73	0.58	0.40	Waist
	**0.79**	0.77	0.67	0.57	0.53	Left Thigh
	0.72	**0.76**	0.61	0.40	0.31	Right Thigh
	0.51	**0.55**	0.62	0.31	0.26	Upper Arm
	0.67	**0.71**	0.56	0.47	0.45	Wrist

**Table 5:** Performance metrics of the selected classifiers for activity recognition at five body positions.

Classifier	Average Accuracy %	Kappa	F-Measure	MAE	RMSE	Body Position
Decision Tree	96.23	0.99	0.96	0.012	0.111	Waist
K-NN	92.53	0.91	0.92	0.025	0.157	
Bayes Net	97.55	0.97	0.97	0.008	0.088	
SVM	99.71	1.00	0.99	0.222	0.310	
Decision Tree	98.90	0.98	0.99	0.004	0.067	Left Thigh
K-NN	95.23	0.94	0.95	0.016	0.125	
Bayes Net	98.57	0.98	0.98	0.005	0.061	
SVM	99.81	1.00	1.00	0.222	0.310	
Decision Tree	97.87	0.97	0.98	0.007	0.083	Right Thigh
K-NN	95.23	0.94	0.95	0.016	0.125	
Bayes Net	98.01	0.97	0.98	0.006	0.080	
SVM	99.47	0.99	0.99	0.222	0.310	
Decision Tree	95.93	0.95	0.96	0.014	0.121	Upper Arm
K-NN	92.58	0.91	0.95	0.025	0.157	
Bayes Net	95.45	0.94	0.95	0.015	0.115	
SVM	98.75	0.98	0.99	0.222	0.310	
Decision Tree	95.18	0.94	0.95	0.017	0.124	Wrist
K-NN	90.93	0.89	0.91	0.031	0.173	
Bayes Net	96.85	0.96	0.97	0.015	0.100	
SVM	98.18	0.97	0.98	0.222	0.311	

**Table 6:** Average performance metrics of the selected classifiers for activity recognition.

Classifier	Average Accuracy %	Kappa	F-Measure	MAE	RMSE
Decision Tree	96.82	0.96	0.96	0.010	0.102
K-NN	93.30	0.91	0.93	0.022	0.147
Bayes Net	97.38	0.96	0.97	0.027	0.086
SVM	99.18	0.98	0.99	0.222	0.310

**Table 7:** Distribution of different users amongst three folds for user classification.

Scenario	No. of Users in Fold-1	No. of Users in Fold-2	No. of Users in Fold-3
A	2	4	4
B	2	3	5
C	3	3	4
D	3	4	3
E	4	3	3

**Table 8:** Results of user classification based on activity recognition at five body positions.

User Class	TPR	FPR	Precision	Recall	F-Measure	Body Position
Authenticated	0.90	0.04	0.90	0.90	0.90	Waist
Supplementary	0.91	0.03	0.92	0.91	0.91	
Impostor	0.95	0.04	0.93	0.95	0.94	
Authenticated	0.92	0.04	0.90	0.91	0.90	Left Thigh
Supplementary	0.90	0.03	0.92	0.90	0.91	
Impostor	0.91	0.05	0.91	0.90	0.91	
Authenticated	0.90	0.04	0.89	0.90	0.90	Right Thigh
Supplementary	0.88	0.04	0.89	0.88	0.88	
Impostor	0.91	0.06	0.90	0.91	0.90	
Authenticated	0.85	0.06	0.86	0.85	0.85	Upper Arm
Supplementary	0.86	0.06	0.85	0.86	0.86	
Impostor	0.86	0.09	0.86	0.86	0.86	
Authenticated	0.82	0.07	0.83	0.82	0.82	Wrist
Supplementary	0.83	0.06	0.85	0.83	0.84	
Impostor	0.90	0.09	0.86	0.90	0.88	
